# Near-Real-Time Acoustic Monitoring of Beaked Whales and Other Cetaceans Using a Seaglider™

**DOI:** 10.1371/journal.pone.0036128

**Published:** 2012-05-18

**Authors:** Holger Klinck, David K. Mellinger, Karolin Klinck, Neil M. Bogue, James C. Luby, William A. Jump, Geoffrey B. Shilling, Trina Litchendorf, Angela S. Wood, Gregory S. Schorr, Robin W. Baird

**Affiliations:** 1 NOAA Pacific Marine Environmental Laboratory, Hatfield Marine Science Center, Cooperative Institute for Marine Resources Studies, Oregon State University, Newport, Oregon, United States of America; 2 Applied Physics Laboratory, University of Washington, Seattle, Washington, United States of America; 3 Cascadia Research Collective, Olympia, Washington, United States of America; Texas A&M University-Corpus Christi, United States of America

## Abstract

In most areas, estimating the presence and distribution of cryptic marine mammal species, such as beaked whales, is extremely difficult using traditional observational techniques such as ship-based visual line transect surveys. Because acoustic methods permit detection of animals underwater, at night, and in poor weather conditions, passive acoustic observation has been used increasingly often over the last decade to study marine mammal distribution, abundance, and movements, as well as for mitigation of potentially harmful anthropogenic effects. However, there is demand for new, cost-effective tools that allow scientists to monitor areas of interest autonomously with high temporal and spatial resolution in near-real time. Here we describe an autonomous underwater vehicle – a glider – equipped with an acoustic sensor and onboard data processing capabilities to passively scan an area for marine mammals in near-real time. The glider was tested extensively off the west coast of the Island of Hawai'i, USA. The instrument covered approximately 390 km during three weeks at sea and collected a total of 194 h of acoustic data. Detections of beaked whales were successfully reported to shore in near-real time. Manual analysis of the recorded data revealed a high number of vocalizations of delphinids and sperm whales. Furthermore, the glider collected vocalizations of unknown origin very similar to those made by known species of beaked whales. The instrument developed here can be used to cost-effectively screen areas of interest for marine mammals for several months at a time. The near-real-time detection and reporting capabilities of the glider can help to protect marine mammals during potentially harmful anthropogenic activities such as seismic exploration for sub-sea fossil fuels or naval sonar exercises. Furthermore, the glider is capable of under-ice operation, allowing investigation of otherwise inaccessible polar environments that are critical habitats for many endangered marine mammal species.

## Introduction

Beaked whales lead a stealthy life in the deep ocean. On their search for prey in deep waters, they dive up to 2000 m and spend as much as 90 min submerged [1], [2]. For these reasons, in most areas it is extremely difficult to investigate beaked whales with traditional visual observing techniques. Only two decades ago, little was known about the family Ziphiidae, which comprises at least 22 species [3]. After a series of beaked whale stranding events associated with naval sonar exercises [4], beaked whales became a research focus in marine mammal science. U.S. environmental laws, including the Endangered Species Act, the Marine Mammal Protection Act, and the National Environmental Policy Act, as well as intense public concern, require the U.S. Navy to conduct its research and operations in a fashion that minimizes impacts on marine mammals, and mitigates any adverse impacts of those operations. As a consequence, the U.S. Navy accelerated research on beaked whales by funding a wide variety of projects to investigate the hearing, vocal behavior, and movements of beaked whales, evaluate adverse effects of man-made sound on them, and develop effective tools to acoustically monitor and protect them during the course of naval exercises [5].

In late 2007, the Applied Physics Laboratory at the University of Washington (APL-UW) and Oregon State University (OSU) started a collaborative research project to develop and use underwater gliders that would autonomously search the ocean for vocalizing beaked whales and report their presence back to shore in near-real time. Underwater gliders use small changes in buoyancy to effect vertical motion, and wings to convert the vertical motion to horizontal movement, thereby propelling themselves forward with very low power consumption. This allows them to perform long-duration surveys autonomously [6]. During a mission, a glider is piloted remotely from a control center onshore. The glider used in this project was the Seaglider™, originally developed by APL-UW [7], which is capable of repeatedly diving to 1000 m depth and back at a typical horizontal speed of 25 cm s^−1^.

Beaked whales vocalize regularly underwater for navigation, prey detection, and potentially communication [8]. The Seaglider used in this study was equipped with an acoustic sensor (hydrophone) to passively listen and detect individuals or groups of animals. A successful proof-of-concept study to record marine mammal vocalizations using a glider was conducted prior to this project [9]. Acoustic methods have been used increasingly often over the last decades to study marine mammal distribution, abundance, and movements, as well as for mitigation of harm to marine mammals [10], [11]. This is due, in part, to the greater availability of the necessary hardware and software and due also to some perceived advantages: the ability to detect animals underwater, to work at night and in poor weather conditions, and to record the relevant signals and post-process them if necessary. Tagging studies revealed that beaked whales predominantly emit sounds at depths greater than 400 m [1]. Because the probability of detection increases with sensor depth [12], deep-diving platforms, including gliders, are well suited to the investigation of these animals.

In fall 2009 APL-UW and OSU conducted a comprehensive glider field test off the west coast of the Island of Hawai'i. Concurrently, scientists from Cascadia Research Collective (CRC) tagged beaked whales with remotely-deployed dorsal-fin attached satellite location tags [13] to monitor large-scale movements of the animals in the area [14]. Here we present the first results of this glider field trial and utilize available tag data to evaluate the performance of the system.

## Methods

### Mission details

**Figure 1:**
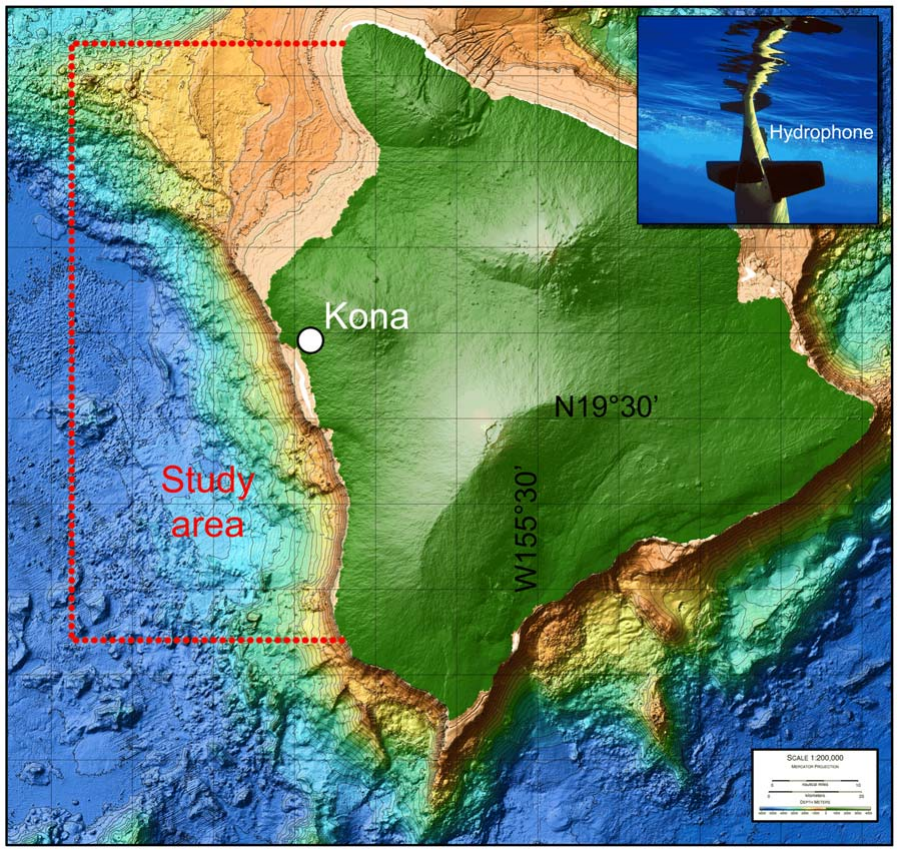
Map of the study area off the Kona coast, Hawai'i, USA. Inset at upper right shows the Seaglider at the beginning of a dive. Bathymetric map source: Hawai'i Mapping Research Group, School of Ocean and Earth Sciences and Technology, University of Hawai'i, USA.

Between 27 October and 17 November 2009, a Seaglider (commercially available from iRobot Corporation, Bedford, MA, USA) equipped with a custom-designed and -built passive acoustic recording system (APL-UW, Seattle, WA, USA) surveyed the west coast of the Island of Hawai'i (see Fig. 1). The primary goal was to detect echolocation clicks of Blainville's (*Mesoplodon densirostris*) and Cuvier's (*Ziphius cavirostris*) beaked whales in near-real time and to report their presence back to shore.

The Seaglider was programmed to operate along a prescribed track between the 1000 m and 2000 m isobaths along the coast, based on previous studies [15] that reported highest beaked whale densities for this area. The glider repeatedly dove to 1000 m depth (or to near the bottom, in water shallower than this) and then ascended to the surface again. The passive-acoustic system was operated at depths below 500 m during 85 glider dives. Acoustic signals were received by a single omni-directional hydrophone (type: HTI-99-HF, High Tech Inc, Gulfport, MS, USA; sensitivity: −164 dB re. 1 V/µPa), amplified by 36 dB, and recorded at 194 kHz sample rate and 16-bit resolution. Acoustic data were compressed using the Free Lossless Audio Codec (FLAC; http://flac.sourceforge.net) and stored on flash memory drives. In parallel, the acoustic data stream was screened in real time onboard for beaked whale vocalizations using the ERMA detection algorithm described earlier [16]. This detector has been proven in an independent study to reliably detect beaked whale echolocation clicks [17]. The Seaglider was operated remotely via Iridium™ satellite communication and configured to report detection events back to shore when surfacing between dives.

### Acoustic data analysis

After recovery of the Seaglider, the entire recorded data set was manually screened by an experienced data analyst (KK) for beaked whale (family Ziphiidae), delphinid (family Delphinidae), and sperm whale (*Physeter macrocephalus*) echolocation clicks, as well as echosounder signals. The result of the “blind" manual beaked whale screening (information on detector output as well as locations of tagged whales were not provided to the analyst) was also used to verify the detections of the onboard real-time detection algorithm. The analysis was done using Matlab™-based analysis software [18] to visualize and annotate acoustic data sets. When searching for time periods with vocal activity, consecutive sound files of 1 minute duration (high-pass filtered at 5 kHz) were screened visually by the analyst using the following spectrogram parameters: frame size and FFT size 2048 samples (11 ms), overlap 50% (5.5 ms), and a Hamming window, for a spectrum filter bandwidth of 385 Hz.

To be able to differentiate beaked whale and delphinid echolocation clicks, a frame size of 32 samples (0.16 ms), FFT size of 128 samples (0.64 ms), overlap 94% (0.15 ms), and Hamming window, for a spectrum filter bandwidth of 24.6 kHz, was used in a second step to resolve the up-sweep frequency contour characteristic of beaked whale echolocation clicks.

Beaked whale echolocation clicks were identified by investigating the clicks' waveform, spectrogram, and spectrum. The following criteria were used for verification: duration, low-frequency roll-off, and frequency modulation (up-sweep) of clicks, as well as the inter-click-interval (ICI) between consecutive clicks. Only three species of beaked whales have been recorded in the surveyed areas, and only two of these are commonly seen: Blainville's and Cuvier's beaked whales [14], [15], [19]. The vocalizations of these beaked whales are well studied and have been described by several authors [8]. Table 1 provides an overview of the principal acoustic features and differences of echolocation clicks produced by the two beaked whale species used by the analyst to distinguish these species.

**Table 1:** Principal acoustic features of echolocation clicks of regular click trains produced by Blainville's (*Mesoplodon densirostris*) and Cuvier's (*Ziphius cavirostris*) beaked whales.

*Species*	*Duration*	*−20 dB low freq. roll-off*	*Inter-click interval*	*Upsweep*
Blainville's beaked whale	250 µs	25 KHz	0.2–0.4 s	yes
Cuvier's beaked whale	175 µs	20 kHz	0.4 s	yes

Values are based on Johnson *et al.*, 2004.

As shown in Table 1, echolocation clicks produced by Blainville's and Cuvier's beaked whales differ significantly in several characteristics. Blainville's beaked whale clicks are longer in duration and feature a higher low-frequency roll-off (20 dB below peak amplitude at 25 kHz). The range of ICIs of Blainville's echolocation clicks is broader (0.2–0.4 s) and more variable than ICIs measured for Cuvier's beaked whale echolocation click trains.

### Tag data analysis

Additionally, a comparison was conducted of tag data collected by CRC and the glider tracks. A potential encounter was identified when (a) at any given time the position of the Seaglider and the surfacing position of a beaked whale were within 6 km, and (b) the accuracy of the reported satellite (ARGOS) tag location was 1.5 km or less. The upper theoretical limit of acoustic detection distance for beaked whales is in the range of 4 km [12]; however, because of the limited accuracy of the locations provided by the tag, a maximum range of 6 km was used.

## Results

### Acoustic data analysis

During the three-week mission, the glider covered approximately 390 km and collected a total of 194 h of acoustic data (11,615 sound files of one minute duration) during 85 glider dives. Average dive duration was 4.3±1.3 hours, with 16.2±12.8 minutes spent at the surface between dives for data transmission. During the mission the Seaglider detected and reported beaked whale vocalizations on 10 out of 85 dives. Manual analysis revealed that 7 of these detections were actual beaked whale encounters. During the other 3 glider dives the detection system was mistakenly triggered by delphinid vocalizations. The analyst identified a total of 109 sound files containing beaked whale clicks. The automated system correctly detected calls in 79 out of these 109 sound files (72%).

**Figure 2:**
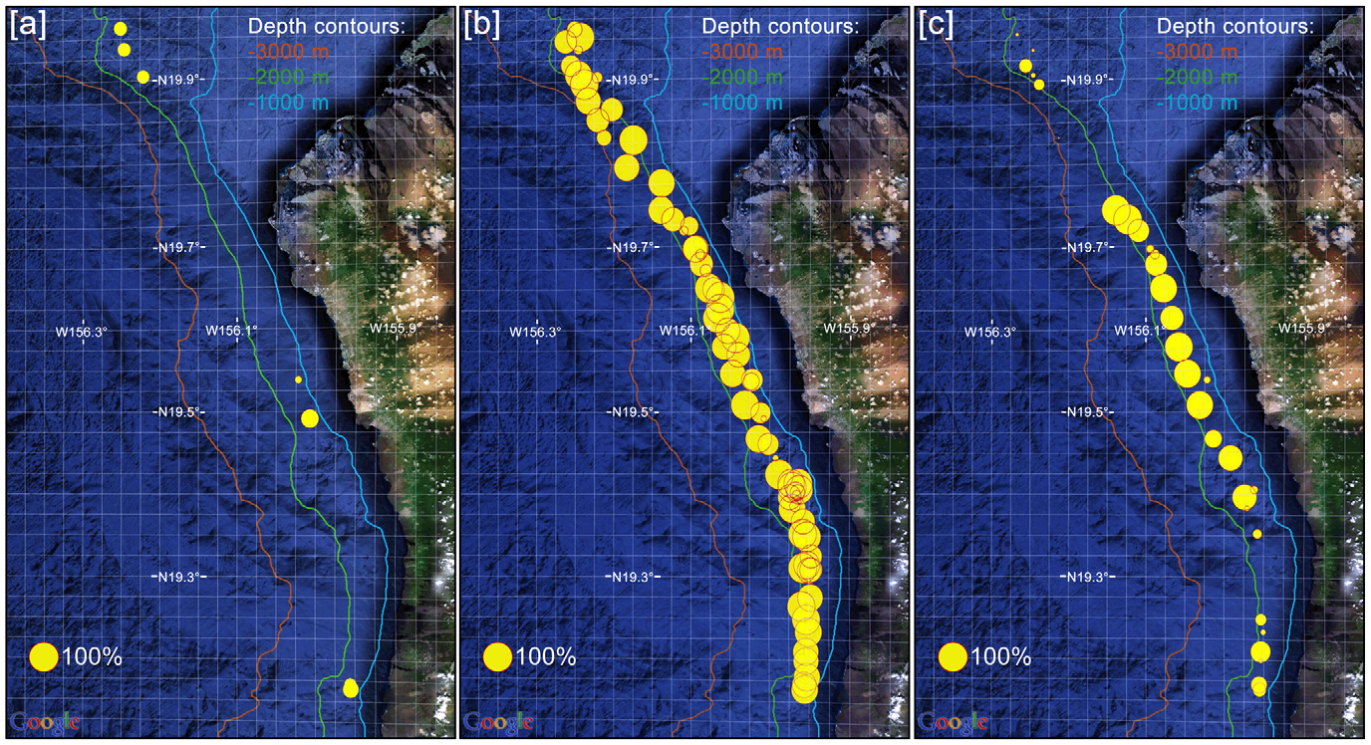
Locations of acoustic encounters as derived from the manual data analysis. Panels indicate locations of [a] beaked whale, [b] delphinid, and [c] sperm whale acoustic encounters. Size of each dot represents the percentage (logarithmic scale) of acoustic data recorded per glider dive containing respective target signal. Map source: Google Earth. Contours: Hawai'i Mapping Research Group, School of Ocean and Earth Sciences and Technology, University of Hawai'i, USA.

**Figure 3:**
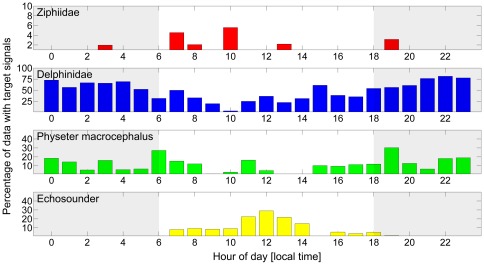
Percentage of data containing target signals in the respective hour of day as derived from manual data analysis. The mean observation duration per hour of day was 484±129 minutes (range 250–696 min). Shaded areas indicate hours before/after sunset. Note different scales of y-axes.

In total 1% of the recorded data contained beaked whale clicks, 50.4% delphinid clicks, 11.8% sperm whale clicks, and 6.5% echosounder signals. The results of the manual data analysis are shown in Figs. 2 and 3.

Fig. 2 shows the locations where cetacean vocalizations were recorded. Beaked whales were recorded during 7 out of 85 glider dives (8%). Five acoustic encounters were identified as Blainville's beaked whales, one as Cuvier's beaked whale (19.97°N, 156.19°W), and one as an unidentified beaked whale (19.56°N, 156.28°W). Delphinid vocalizations were the predominant bioacoustic signals in the recordings and were roughly distributed evenly along the track of the glider. Sperm whale vocalizations were recorded primarily along the central west coast of the Island of Hawai'i.

**Figure 4:**
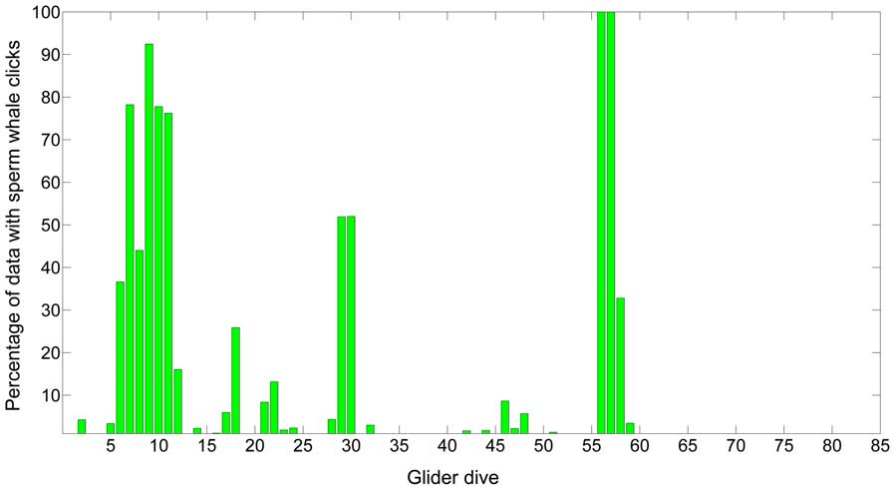
Percentage of manually analyzed acoustic data containing sperm whale vocalizations for respective glider dives.

Fig. 3 shows the percentage of recorded data containing target signals versus hour of day (local time). As expected, echosounder signals (fish finders and depth sounders) were recorded mainly in daytime, when most recreational fishing and boating occurs. Sperm whales were recorded throughout the day, with minimum detection rates during mid-morning (9:00–10:00 local time (LT)) and early afternoon (13:00–14:00 LT). Fig. 4 shows the percentage of recorded data containing sperm whale clicks by glider dive number, and indicates that sperm whales were in the area of the glider primarily on three occasions (one lasting more than a day) and were detected either in smaller numbers or at greater distances on several other occasions.

Delphinid echolocation clicks showed a clear diurnal pattern, with high detection rates during the night (maximum at 22:00 LT) and low rates during the day (minimum at 10:00 LT). At 22:00 LT more than 75% of the recorded data contained delphinid vocalizations. This percentage dropped during the course of the day to below 10% at 10:00 LT. Recordings of beaked whale echolocation clicks were scattered throughout the day with no apparent pattern. A one-way ANOVA analysis was used to statistically verify the pattern described above. The ANOVA analysis confirmed (*p*<0.01) that (a) the number of recorded delphinid vocalizations is significantly higher during night-time, and (b) the number of recorded echosounder signals is significantly higher during daytime. No statistically significant day/night patterns were found for beaked whales and sperm whales.

### Tag data analysis

**Figure 5:**
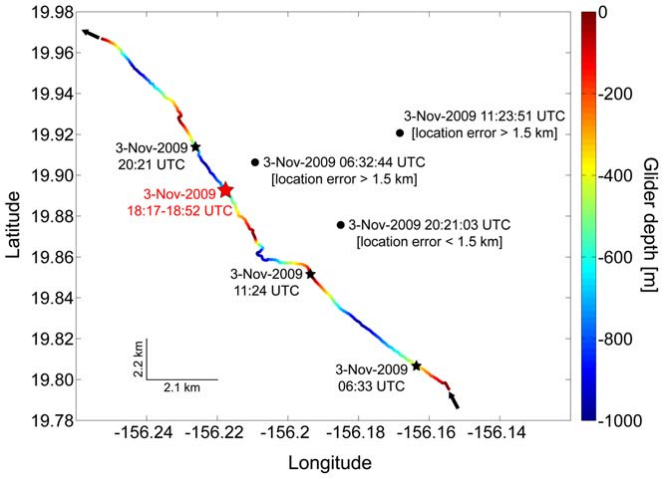
Glider track (colored line) and surfacing positions of tagged Cuvier's beaked whale (black dots) on 3 November 2009. Glider depth is color-coded. Black stars indicate position of glider at times of beaked whale surfacing events. Red star indicates position of glider when Cuvier's beaked whale clicks were acoustically detected by the glider during the mission (detections were verified in the post deployment analysis). The acoustic system was operated at depth between 500 m and 1000 m indicated by greenish/bluish colors. Times are UTC.

The comparison of available tag data and the glider track revealed one potential encounter with a tagged Cuvier's beaked whale. The potential encounter occurred on 3 November 2009 and covered four glider dives with a total of approximately 12 hours of recorded data. Fig. 5 provides an overview of the beaked whale surfacing positions and the glider track. The glider was travelling SSE to NNW when the tagged beaked whale surfaced east of the glider at a distance between 6 and 12 km.

Acoustic data analysis confirmed one acoustic encounter as reported by the glider during the mission with Cuvier's beaked whales on 3 November 2009. The acoustic encounter (18:17–18:52 UTC) occurred shortly before the surfacing event (20:21 UTC).

## Discussion

Manual analysis of the recorded data revealed a high degree of bioacoustic activity. Delphinids and sperm whales produced the majority of recorded vocalizations. The observed diurnal pattern in recorded delphinid vocalizations is likely caused by two behavioral patterns.

- Diel activity pattern: The most frequently encountered species of delphinids in the area (Baird, unpublished data), namely short-finned pilot whales (*Globicephala macrorhynchus*) and pantropical spotted dolphins (*Stenella attenuata*), both show increased foraging activity, and thus likely echolocation activity, at night [20], [21]. Furthermore, several other species of delphinids in the study area (e.g., melon-headed whales) are known to rest or travel primarily during the day [22], [23].
- Diel migration: Spinner dolphins (*Stenella longirostris*) show a diurnal migration pattern, remaining in shallow near-shore water during the day and moving offshore at night [24], although they do move closer to shore in the middle of the night following the migration of their prey into shallow water [25]. According to Benoit-Bird and Au [25], offshore echolocation activity by spinner dolphins occurs during late evening (∼21:00 LT) and early morning (∼03:00 LT), with a dip around midnight. The results of the analysis of the glider data indicate slightly higher delphinid vocalization rates at around 22:00 and 04:00 LT with a dip at around 01:00 LT, results which overall match previous findings [25].

Accordingly, the observed diel pattern in delphind vocal activity is likely associated with at least two behavioral patterns by different species. The acoustic data set collected over the 100 km of coastline traversed during this glider survey suggests that the observed pattern probably occurs along the entire west coast of the Island of Hawai'i.

Sperm whales were detected during approximately 12% of the total recordings, although the temporal clustering of detections suggested they were in the area on about eight different occasions, with the majority of detections recorded primarily on three occasions (Fig. 4). Such a high frequency of detection of sperm whales is somewhat surprising: during visual surveys in the area, sperm whales are seen less frequently than either Cuvier's or Blainville's beaked whales (Baird, unpublished data), yet are more easily detected from a distance than either species. The large number of acoustic detections of sperm whales relative to beaked whales likely reflects larger average group sizes, clicking behavior that occurs during a greater proportion of the time, and a greater average distance that sperm whale clicks can be detected compared to beaked whale clicks.

Beaked whales were recorded during 7 out of 85 glider dives, or one acoustic encounter every 27.7 hours of recording. This is a similar rate of encounters for beaked whales detected visually in small-boat surveys off the island of Hawai'i, with one encounter every 26.8 hours of visual survey effort between 500 and 4000 m depth (Baird, unpublished data). All seven acoustic beaked whale encounters were noted by the real-time detection system and reported to shore during the mission. However, the system also reported three false positive detections triggered by vocalizing delphinids. To improve the detection performance of the system, a second-stage classifier [26] is currently being implemented on the Seaglider. Furthermore, the glider now features the capability of transferring selected acoustic data snippets via Iridium satellite communication during a mission for manual verification.

**Figure 6:**
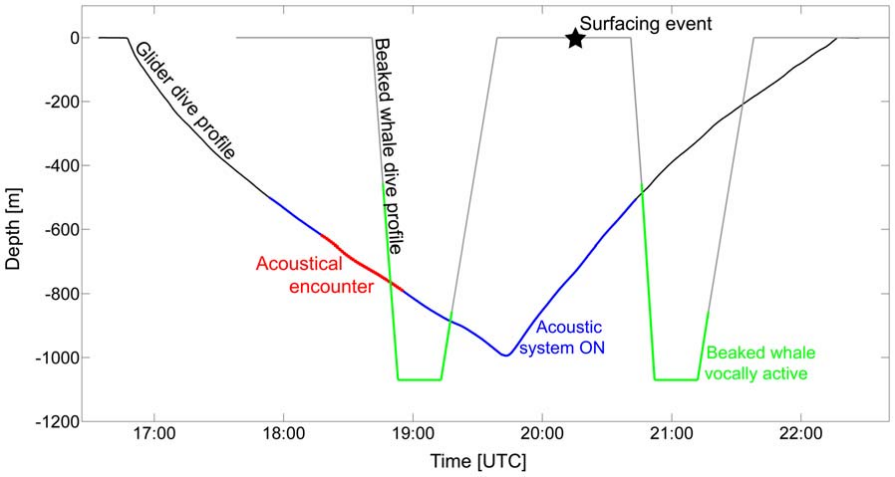
Glider track (v-shaped line) and hypothetical dive profile of tagged Cuvier's beaked whale (u-shaped line); see text for details. **Blue line indicates the time/depth when the acoustic system of the glider operated.** Red line represents time/depth when Cuvier's beaked whales were acoustically detected by the glider. Green line marks periods when the whale was presumably vocally active. Black star indicates surfacing position of the whale. Remarks: [1] This graph does not consider horizontal distances and the orientation of the whale towards the glider. [2] For illustration purposes, the whale's dive profile was limited to deep dives only.

A comparison of the acoustic data collected and available tag data revealed a potential encounter between a Cuvier's beaked whale and the glider on 3 November 2009. Cuvier's beaked whale echolocation clicks were recorded by the glider at 18:17–18:52 UTC approximately 2 hours prior to a tagged Cuvier's beaked whale surfacing event at 20:21 UTC. At the time of surfacing, the distance between whale and glider was 5.8 km (±1.5 km). Assuming the whale dove in the close vicinity of its surfacing position, it is likely that the glider was located even closer to the whale at the time of the acoustic encounter. Although the exact distance between whale and glider could not be determined, the data confirmed that the glider is capable of detecting the presence of vocalizing beaked whales at a few kilometers distance. Interestingly, the glider failed to register echolocation clicks of the tagged beaked whale after the surfacing event. A possible explanation could be the intermittent sampling scheme of the glider: the glider track, acoustic recording times, and a hypothesized dive profile of the tagged Cuvier's beaked whale are shown in Fig. 6.

The hypothesized whale dive profile was generated by applying mean dive parameters for Cuvier's beaked whales [1], and aligning the satellite transmission at the centre of the surface period. Cuvier's beaked whales are known to perform shallow dives (<400 m) in between deep foraging dives [1], [15]. As the whales remain silent during these dives [1], the graph was simplified and shows only deep dives. Fig. 6 illustrates the fact that the first descending dive of the glider and beaked whale potentially overlapped temporally. This would explain the extended period of acoustic detections (18:17–18:52 UTC) indicated by the red line. The entire second dive of the Cuvier's beaked whale possibly occurred during a period when the acoustic system onboard the glider was offline (solid black line) and would explain why the glider didn't register echolocation clicks after the surfacing event. This result is crucial for planning future glider operations. To minimize the number of missed encounters, the acoustic system of the glider should be turned on a greater proportion of the time, say at depths lower than 100 m. As an alternative, multiple gliders could be operated in the same area with alternating dive times; however, this would increase the complexity of operations as well as the overall cost.

**Figure 7:**
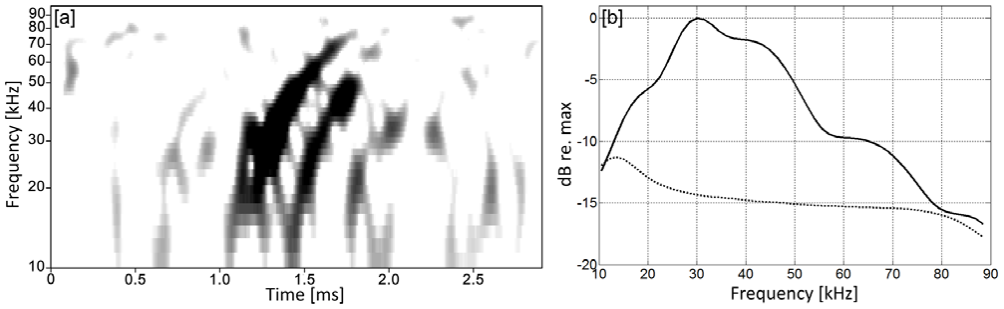
Example of unknown echolocation clicks likely produced by beaked whales. Figure 7a shows a spectrogram of the ‘double click’. Recording was made at 500 m depth. Figure 7b represents a spectrum of the click (solid line) and the background noise level at the time of the recording (dotted line). Spectrogram parameters used to generate plots: frame size 32 samples (0.16 ms), FFT size 128 samples (0.64 ms), overlap 94% (0.15 ms), and Hamming window, for a spectrum filter bandwidth of 24.6 kHz. Data were high-pass filtered at 10 kHz prior to processing.

During the mission, the glider also registered unknown echolocation clicks. Whereas the click spectrogram (Fig. 7a) revealed the upsweep contour characteristic of beaked whales, and the low-frequency roll-off of the spectrum matched that of other beaked whales, the clicks did not match the acoustic features of known beaked whale clicks as described in literature. The glider registered only a single click train, consisting of 11 clicks, of this unidentified species. The low number of recorded clicks as well as the low band-limited (15–90 kHz) signal-to-noise ratio (SNR_0-p_) of 5.7±1.4 dB prevented extraction of statistically significant acoustic features. However, the ICI between the clicks was significantly shorter (0.12±0.01 s) than for the previously reported Blainville's and Cuvier's beaked whale [8], Longman's beaked whale [27], and an unidentified beaked whale species recorded offshore Hawai'i at Cross Seamount [28], [29]. Although the SNR of these recorded clicks was relatively low, reducing the accuracy of measurements, the clicks seemed to be comparatively long in duration (530±95 µs). The clicks covered the frequency range 15–80 kHz and the peak frequency was approximately 30 kHz. It is unknown what caused the ‘double click feature’ as shown in the spectrogram in Fig. 7a. Considering the recording and ocean depth, a reflection close to the glider from the sea surface or bottom can be excluded as the cause. Although the second click (which was apparent for all clicks in the click train) could potentially originate from a vehicle surface bounce, the ‘double click feature’ has never been observed for any other echolocations clicks recorded with the Seaglider.

In conclusion, this study shows that passive acoustic gliders have significant potential as platforms for monitoring marine mammals. These autonomous instruments can be remotely operated from shore for several months at a time and permit cost-effective continuous monitoring of marine mammals independently of weather and light conditions. Although this study concentrated on high-frequency cetaceans, gliders can also be used to monitor low-frequency cetaceans such as baleen whales [30]. The most advanced feature of the system described here is its near-real-time detecting/messaging capability, which is useful for time-critical applications in the context of mitigating injury to, or mortality of, cetaceans during anthropogenic activities such as naval exercises or seismic oil and gas exploration. In addition, the glider is by default equipped with a conductivity-temperature-depth (CTD) sensor, providing useful information on oceanographic conditions in the survey area. Furthermore, add-on sensors (e.g., O_2_, pH) can be used to deploy multi-sensor platforms to investigate broader scientific questions associated with marine mammals. Finally, RAFOS equipped Seagliders are capable of under-ice operation, allowing scientists to investigate polar environments containing critical habitats for many endangered marine mammal species.
