# Correction: Near-Real-Time Acoustic Monitoring of Beaked Whales and Other Cetaceans Using a Seaglider™

**DOI:** 10.1371/annotation/57ad0b82-87c4-472d-b90b-b9c6f84947f8

**Published:** 2012-08-10

**Authors:** Holger Klinck, David K. Mellinger, Karolin Klinck, Neil M. Bogue, James C. Luby, William A. Jump, Geoffrey B. Shilling, Trina Litchendorf, Angela S. Wood, Gregory S. Schorr, Robin W. Baird

There was an error in affiliation 1 for authors Holger Klinck, David K. Mellinger, and Karolin Klinck. Affiliation 1 should be: Cooperative Institute for Marine Resources Studies, Oregon State University and NOAA Pacific Marine Environmental Laboratory, Hatfield Marine Science Center, Newport, Oregon, United States of America

